# Inactivating Mutations of the *IK* Gene Weaken Ku80/Ku70-Mediated DNA Repair and Sensitize Endometrial Cancer to Chemotherapy

**DOI:** 10.3390/cancers13102487

**Published:** 2021-05-20

**Authors:** Chao Gao, Guangxu Jin, Elizabeth Forbes, Lingegowda S. Mangala, Yingmei Wang, Cristian Rodriguez-Aguayo, Paola Amero, Emine Bayraktar, Ye Yan, Gabriel Lopez-Berestein, Russell R. Broaddus, Anil K. Sood, Fengxia Xue, Wei Zhang

**Affiliations:** 1 Department of Cancer Biology, Wake Forest Baptist Comprehensive Cancer Center, Winston-Salem, NC 27157, USA; chaogao1224@tmu.edu.cn (C.G.); gjin@wakehealth.edu (G.J.); eforbes@wakehealth.edu (E.F.); 2 Department of Gynecology and Obstetrics, Tianjin Medical University General Hospital, Tianjin 300052, China; wangyingmei@tmu.edu.cn (Y.W.); yanye@tmu.edu.cn (Y.Y.); 3 Tianjin Key Laboratory of Female Reproductive Health and Eugenics, Tianjin 300052, China; 4 Department of Gynecologic Oncology and Reproductive Medicine, The University of Texas M.D. Anderson Cancer Center, Houston, TX 77030, USA; lsmangala@mdanderson.org (L.S.M.); EBayraktar@mdanderson.org (E.B.); asood@mdanderson.org (A.K.S.); 5 Center for RNA Interference and Non-Coding RNAs, The University of Texas M.D. Anderson Cancer Center, Houston, TX 77030, USA; CRodriguez2@mdanderson.org (C.R.-A.); glopez@mdanderson.org (G.L.-B.); 6 Department of Experimental Therapeutics, The University of Texas M.D. Anderson Cancer Center, Houston, TX 77030, USA; pamero@mdanderson.org; 7 Department of Pathology & Laboratory Medicine, University of North Carolina School of Medicine, Chapel Hill, NC 27599, USA; rbroaddus@med.unc.edu

**Keywords:** IK, somatic mutation, DNA repair, endometrial cancer, Ku80, cisplatin

## Abstract

**Simple Summary:**

Some endometrial cancer (EC) patients have different prognoses and responses to treatment, even among those with the same stage and tumor grade as assessed by the International Federation of Gynecology and Obstetrics (FIGO) criteria. Molecular classification may make up for this deficiency to some extent. Our previous investigation of the 271 EC samples from the Cancer Genome Atlas (TCGA) dataset showed that IK somatic mutations were enriched in a cluster of patients with high-grade and high-stage cancers, and this group had longer overall survival. This current study continued to provide insight into how IK somatic mutations contribute to EC pathophysiology. We explored IK gene mutations in depth and used functional studies to characterize an unrecognized function of the IK gene in EC. Our findings may elucidate the molecular mechanism of IK in EC, which might guide future patient stratification and contribute targeted therapy for EC.

**Abstract:**

IK is a mitotic factor that promotes cell cycle progression. Our previous investigation of 271 endometrial cancer (EC) samples from the Cancer Genome Atlas (TCGA) dataset showed IK somatic mutations were enriched in a cluster of patients with high-grade and high-stage cancers, and this group had longer survival. This study provides insight into how IK somatic mutations contribute to EC pathophysiology. We analyzed the somatic mutational landscape of IK gene in 547 EC patients using expanded TCGA dataset. Co-immunoprecipitation and mass spectrometry were used to identify protein interactions. In vitro and in vivo experiments were used to evaluate IK’s role in EC. The patients with IK-inactivating mutations had longer survival during 10-year follow-up. Frameshift and stop-gain were common mutations and were associated with decreased IK expression. IK knockdown led to enrichment of G2/M phase cells, inactivation of DNA repair signaling mediated by heterodimerization of Ku80 and Ku70, and sensitization of EC cells to cisplatin treatment. IK/Ku80 mutations were accompanied by higher mutation rates and associated with significantly better overall survival. Inactivating mutations of IK gene and loss of IK protein expression were associated with weakened Ku80/Ku70-mediated DNA repair, increased mutation burden, and better response to chemotherapy in patients with EC.

## 1. Introduction

Endometrial cancer (EC) is one of the three most prevalent gynecologic malignancies, and its incidence and mortality have risen in the last few decades [1,2]. Compared with the other two types of gynecologic malignancies, EC has a relatively better prognosis. However, those with EC have different prognoses and responses to treatment, even among those with the same stage and tumor grade as assessed by the International Federation of Gynecology and Obstetrics (FIGO) criteria [3]. Hence, molecular characterization by genomic mutations may be a way to address this heterogeneity. A proactive molecular risk classification tool for endometrial cancers (ProMisE) was developed to improve EC subgroup assignment and risk assessment [4,5]. Based on sequencing and immunohistochemical results, ProMisE includes mismatch repair deficiency (MMRd), DNA polymerase epsilon (*POLE*) mutations, wild-type *p53*, and abnormal *p53* as molecular markers. *POLE* is mutated in many cancers, including pancreatic, ovarian, colorectal, and endometrial cancer [6,7,8,9]. *POLE* mutations are associated with improved prognosis in EC [9].

The TransPORTEC, an international consortium related to the Post Operative Radiation Therapy for Endometrial Cancer (PORTEC) 3 trial, identified four new subgroups of high-risk EC: a *POLE* proofreading mutant group, a *p53* mutant group, a microsatellite instable group, and a group with no specific molecular profile [10]. This new molecular classification of high-risk EC can be effectively used to identify distinct subsets with prognostic significance and supplement traditional FIGO stage and tumor grade classifications. However, other relevant EC markers and gene mutations, such as *CTNNB1* [11] and *ARID1A* [12], may also affect molecular subtyping.

IK is a mitotic factor that regulates cell cycle progression and proliferation. It was coded by the *IK* gene, which was mapped to chromosome 5q22-q23 [13]. IK was first isolated and purified from the culture medium of the human leukemic cell line K562 in 1987 [14]. This type of secretory IK was a truncated form of IK (tIK) with protein size 19 kDa. The full-length IK is a nuclear protein consisting of 557 amino acids with protein size 65 kDa [13,15]. RED is an alternative name for IK due to its protein sequence. An extensive stretch of alternating arginine (R), glutamic acid (E) and (or) aspartic acid (D) residues constitute RED repeats, referring to amino acid residues 334 to 375 [13]. Some studies showed that RED repeats were well-known autoantigens in systemic lupus erythematosus and contributed to form stable beta sheet structures [16,17]. IK was named after its immune-related role in the inhibition of interferon gamma (IFN-γ)-induced expression of human leukocyte antigen (HLA) class II antigen [14,18]. Previous studies showed that IK could bind strongly to protein phosphatase 2A (PP2A), which is involved in cell cycle progression through the regulation of mitotic spindle dynamics and centrosome separation, and that IK depletion decreased cell proliferation and caused cell cycle arrest [19,20]. In addition, *IK* was suggested as a cell death-inducing gene in a previous siRNA library screening [21]. Overall, however, the role of IK in cancer has been poorly investigated.

In our previous study, we found that the *IK* gene was only mutated in Cluster IV of endometrioid endometrial cancer (EEC) samples in the Cancer Genome Atlas (TCGA) cohort [11]. In addition, gene expression profiling of the most common subtype of EC (EEC) led to recognition of four subtypes. Clusters I and II included low-grade and early-stage EC, whereas Clusters III and IV were more likely to include late-stage and high-grade EC. Interestingly, *IK* mutated EC samples in Cluster IV were associated with better survival, even compared to Cluster II. To further understand the important role of *IK* mutation in EC and its downstream effects on cellular functions, we integrated bioinformatics analyses and laboratory experiments to investigate the possible role of *IK* deficiency and gain insight into whether *IK* mutation can be considered as a new marker to classify EC. We evaluated the role of IK in DNA repair and cell cycle regulation to determine the underlying mechanisms that account for the observation that *IK* mutations improve response to chemotherapy and overall survival in patients with EC.

## 2. Results

### 2.1. Somatic Mutations of the IK Gene in EEC

**Figure 1:**
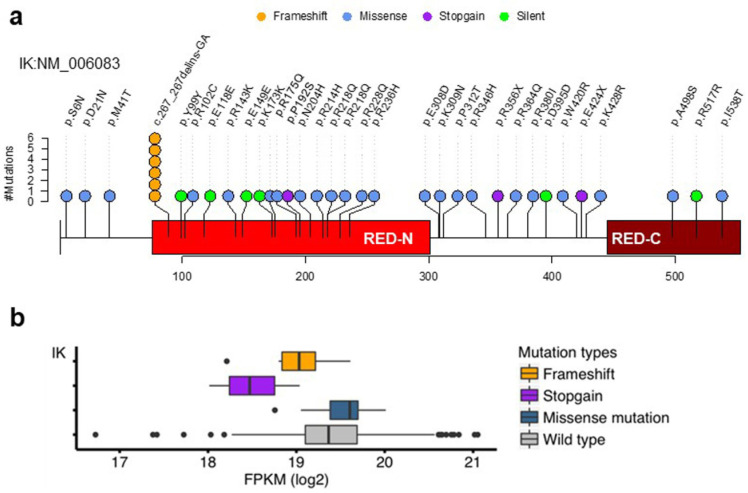
Somatic mutations of the *IK* gene in EEC. (**a**) *IK* somatic mutations. The plot was generated by OncoPrint in cBioPortal (https://www.cbioportal.org/ (accessed on 4 April 2017). (**b**) Comparison of gene expression between patients with somatic mutations of *IK* and those with wild-type *IK*. FPKM, fragments per kilobase of transcript per million mapped reads. *p* = 0.001, Student’s *t* test for frameshift and stop-gain mutations.

We analyzed the cohort of 547 EEC patients in TCGA (Table S1) and identified 38 somatic mutations of the *IK* gene, including six patients with R93fs deletion (c_267_267delines-GA), three patients with stop-gain nonsense mutations, and others with missense mutations (Figure 1a and Table S2). These mutations were identified by using the whole exome sequencing data of 547 EEC samples from Genomics Data Commons (Materials and Methods). To examine whether the R93fs deletion (c_267_267delines-GA) is a germline mutation or an artifact caused by technical errors, we analyzed germline mutations in normal tissues from the 547 EEC patients, fragments of paired-end reads aligned to the GA repetitive adjacent to c_267_267delines-GA, and forward and reverse strands of the aligned reads with the c_267_267delines-GA somatic mutation (see Materials and Methods). We confirmed that R93fs deletion (c_267_267delines-GA) was neither a germline mutation nor an artifact caused by technique errors (e.g., single strand or read orientation). Furthermore, we also used the IGV software (http://software.broadinstitute.org/software/igv/ (accessed on 3 February 2021) to manually verify the somatic mutation by using both tumor and normal bam files.

Consistent with our previous study with a smaller cohort of TCGA EC cases, the *IK* gene mutations in expanded TCGA cohort were highly associated with Grade 3 (G3) EEC (*p* < 2.2 × 10^−16^, Fisher’s exact test) (Table S3). Patients with *IK* mutations survived longer than those without *IK* mutations during 10-year follow-up (Table S4).

Among the mutations identified, frameshift and stop-gain mutations (approximately 28% of the nonsynonymous mutations) were associated with significantly lower levels of *IK* expression (*p* = 0.0014, Student’s *t* test) (Figure 1b).

### 2.2. IK Attenuation Affects Cell Cycle and Sensitizes EC to Cisplatin In Vitro and In Vivo

**Figure 2:**
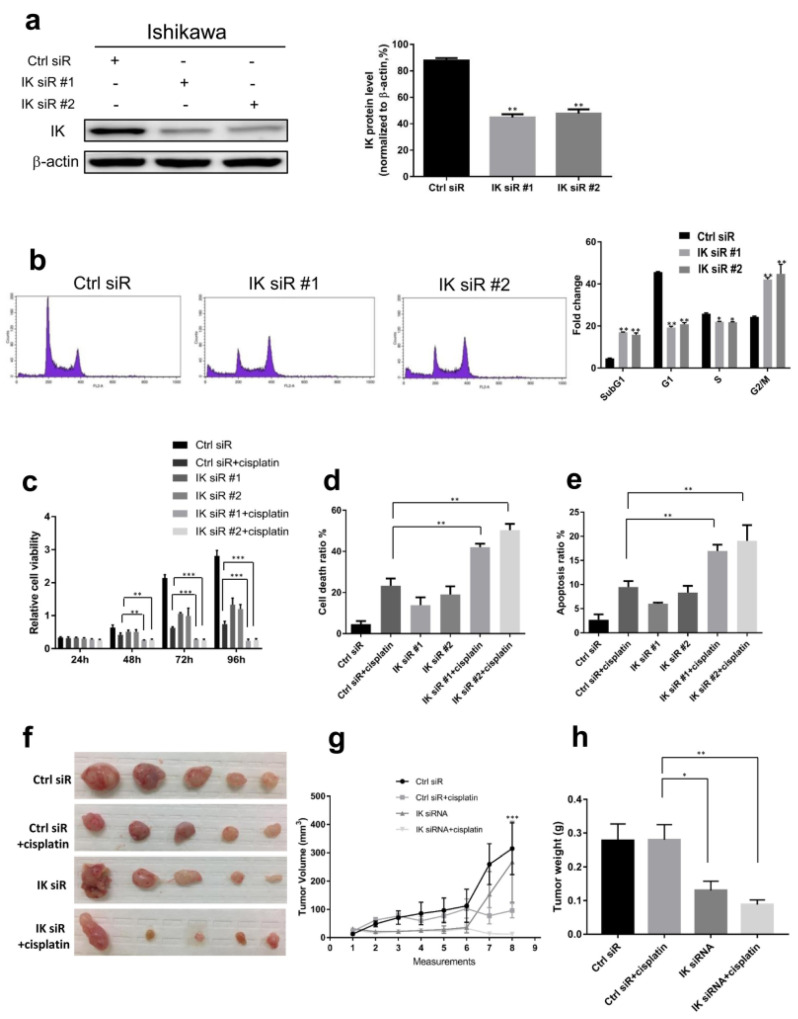
IK attenuation affects cell cycle and sensitizes EC to cisplatin in vitro and in vivo. (**a**) (Left) Seventy-two hours after IK siRNA transfection in Ishikawa cells, IK expression was attenuated. (Right) Quantitative analysis of IK protein expression. (**b**) (Left) Forty-eight hours after IK siRNA transfection, IK attenuation led to enrichment of G2/M cells. (Right) Quantification of cells in different phases. (**c**) CCK-8 assay showed IK attenuation sensitized Ishikawa cells to cisplatin treatment. The IK siRNA transfection plus cisplatin group inhibited cell viability more significantly. (**d**) Seventy-two hours after IK siRNA transfection with or without cisplatin treatment, the IK siRNA transfection plus cisplatin group had more dead cells on trypan blue exclusion assays. (**e**) Seventy-two hours after IK siRNA transfection with or without cisplatin treatment, the IK siRNA transfection plus cisplatin group had more apoptotic cells. (**f**) Representative images of tumors in nude mice treated with control siRNA-DOPC, control siRNA-DOPC plus cisplatin, IK siRNA-DOPC, or IK siRNA-DOPC plus cisplatin (n = 10 per group). Tumor volume (**g**) and tumor weight (**h**) in each group 4 weeks after different treatments. Mean ± SD of at least three independent experiments for in vitro studies (two-sided Student’s *t* test, * *p* < 0.05, ** *p* < 0.01, *** *p* < 0.001).

Since the highly statistically significant association between IK mutations and EC patient survival and the high fraction of loss-of function frameshift and stop-gain mutations, we hypothesized that loss of IK could have a great impact on cellular functions in EC tumor cells. To test this hypothesis, we explored IK functions in vitro and in vivo. We knocked down IK using two different pools of siRNA in the Ishikawa and KLE EC cell lines (*p* < 0.01) (Figure 2a and Figure S1c). After IK siRNA transfection, G2/M phase cells were significantly enriched in Ishikawa cells after 48 and 72 h, but not after 24 h (*p* < 0.01) (Figure 2b and Figure S1a). In KLE cells, IK siRNA transfection did not lead to obvious G2/M phase enrichment (Figure S1b). SubG1 cells, which are apoptotic, increased significantly after IK siRNA transfection in both cell lines (*p* < 0.01) (Figure 2b and Figure S1a,b). Increased cell apoptosis was confirmed using annexin V-FITC/PI staining assays (Figure S2).

Meanwhile, we also found that caspase 3/7, caspase 8, and caspase 9 were activated (*p* < 0.001) (Figure S3a). After the same time period, treatment with Q-VD-OPH, a cell apoptosis inhibitor, decreased cell death ratio measured by trypan blue exclusion assays (*p* < 0.01) (Figure S3b,c).

Platinum-based chemotherapy is a front-line therapy for patients with advanced or relapsed EC [22]. These drugs induce oxidative stress and DNA damage as a mechanism for killing tumor cells [23]. To determine whether *IK* mutation and loss of function contributed to response to chemotherapy and thereby to improved survival, we performed in vitro and in vivo experiments. In the in vitro studies, IK siRNA transfection caused decreased cell viability and cell proliferation (*p* < 0.001) (Figure S4). IK siRNA transfection and cisplatin co-treatment significantly reduced cell viability compared with IK siRNA transfection or cisplatin treatment alone in both EC cell lines (*p* < 0.01) (Figure 2c and Figure S5a), while the cell death ratio and cell apoptosis ratio increased (*p* < 0.01) (Figure 2d,e and Figure S5b,c).

Next, we established an EC xenograft model by transplanting Ishikawa cells into nude mice followed by treatment with IK siRNA-DOPC and/or cisplatin (see Methods). In this model, tumor volume was measured at 8 time points (twice weekly) after injection of cancer cells. Tumors treated with IK siRNA-DOPC grew much more slowly during the first 6 time points than tumors given control siRNA-DOPC (*p* < 0.05), although the difference was less at the latter two time points (*p* = 0.309) (Figure 2g). Tumors treated with IK siRNA-DOPC weighed 53.2% less than those in the control siRNA-DOPC group (*p* < 0.05) (Figure 2h). Tumors co-treated with IK siRNA-DOPC, and cisplatin had significantly decreased tumor volume and weight compared to the IK siRNA-DOPC or cisplatin treatment alone groups (*p* < 0.05) (Figure 2f–h and Figure S6a). Similar but less marked results were observed with the HEC1A model (Figure S6b–e).

### 2.3. IK Interacts with Ku80 and Modulates Ku80 Complexing with Ku70

**Figure 3:**
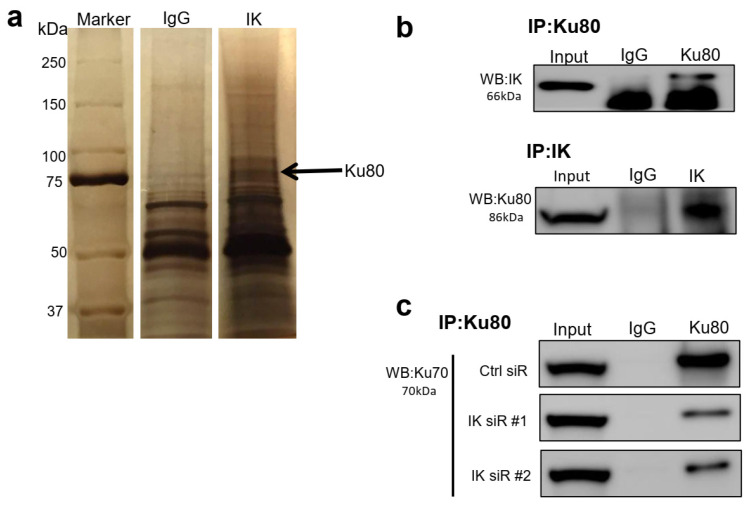
IK interacts with Ku80 and modulates Ku80 complexing with Ku70. (**a**) After DNA-damaging treatment (10 µM cisplatin for 1 h), proteins from IP experiments were analyzed by SDS-PAGE and silver stained. (**b**) After cisplatin treatment, Ku80 directly interacted with IK. (**c**) Seventy-two hours after IK siRNA transfection with cisplatin treatment, IK attenuation weakened Ku80 complexing with Ku70.

Because decreased IK sensitized EC cells to DNA-damaging drugs, we investigated whether IK is involved in DNA repair, especially after cells were challenged by chemotherapy. Ishikawa cells were treated by cisplatin (10 µM for 1 h), the nuclear protein isolated, and subjected to immunoprecipitation (IP) with an IK antibody. IK precipitates were analyzed by mass spectrometry. Among the proteins found in this exploratory analysis was Ku80 (also called X-Ray Repair Cross Complementing 5, XRCC5), a key protein in the non-homologous end joining (NHEJ) DNA repair pathway (Figure 3a and Figure S7). Subsequently, we performed reciprocal IP followed by Western blotting, and validated that IK formed a complex with Ku80 (Figure 3b).

In the NHEJ signaling repair pathway, the heterodimer Ku80/Ku70 recognizes and binds with damaged DNA and then recruits downstream proteins, such as DNA-PKcs [24,25,26]. To examine whether IK attenuation affects endogenous heterodimerization of Ku80 and Ku70, we performed IP assays after transfection of IK siRNA in Ishikawa cells. IK knockdown weakened the ability of Ku80 and Ku70 to interact (Figure 3c).

### 2.4. IK Knockdown Leads to Inactivation of DNA Repair Signaling

**Figure 4:**
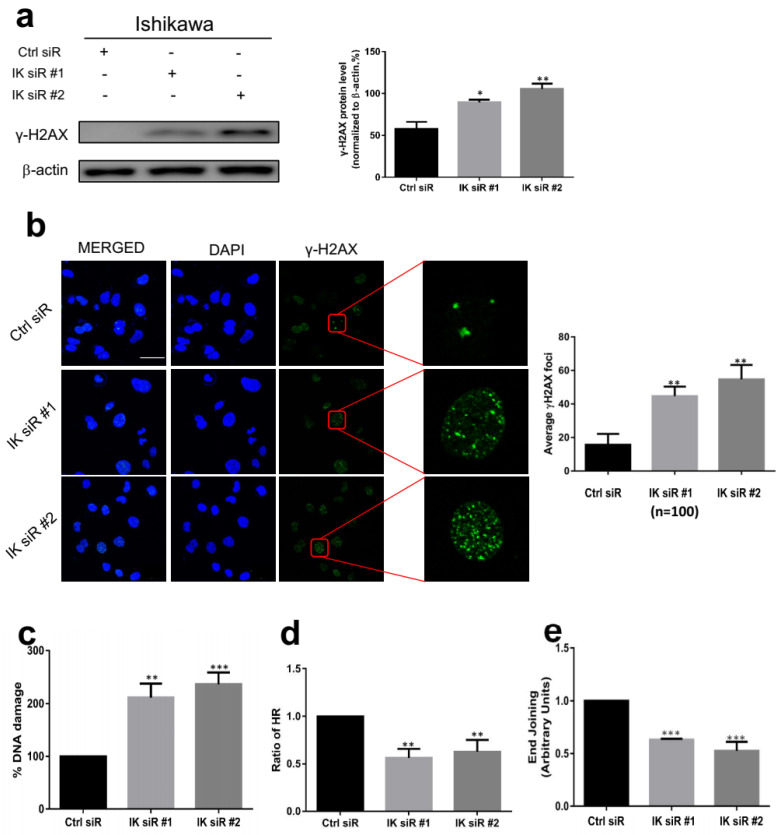
IK knockdown leads to inactivation of DNA repair signaling. (**a**) (Left) Seventy-two hours after IK siRNA transfection in Ishikawa cells, γ-H2AX expression increased. (Right) Quantitative analysis of γ-H2AX protein expression. (**b**) (Left) Seventy-two hours after IK siRNA transfection, IK attenuation caused more γ-H2AX foci. (Right) Quantification of average γ-H2AX foci per cell. (**c**) Seventy-two hours after IK siRNA transfection, IK attenuation caused more DNA damage. (**d**) Seventy-two hours after IK siRNA transfection, IK attenuation weakened HR efficiency. (**e**) Seventy-two hours after IK siRNA transfection, we measured end joining in Ishikawa nuclear extracts of different groups with qPCR; IK attenuation weakened NHEJ efficiency. Mean ± SD of at least three independent experiments (two-sided Student’s *t* test, * *p* < 0.05, ** *p* < 0.01, *** *p* < 0.001).

Interaction of IK with the Ku80/Ku70 DNA repair complex suggested that IK is involved in DNA repair. We subsequently tested this hypothesis. γ-H2AX is a common indicator for DNA damage and damage repair signaling [27]. Seventy-two hours after IK siRNA transfection, γ-H2AX expression increased in Ishikawa and KLE cells on Western blots (*p* < 0.01) (Figure 4a and Figure S8a). Immunofluorescence staining showed that IK siRNA treatment significantly increased the number of γ-H2AX foci (*p* < 0.01) (Figure 4b and Figure S8b). We further quantified DNA damage using EpiQuik In Situ DNA Damage Assays. Seventy-two hours after IK siRNA transfection, treated cells had significantly more DNA damage than the negative control group (*p* < 0.01) (Figure 4c and Figure S8c). Using established assays for NHEJ and homologous recombination (HR)-mediated DNA repair (see Methods), IK siRNA treatment significantly decreased HR and NHEJ capacity (*p* < 0.01) (Figure 4d,e and Figure S8d,e).

### 2.5. Mutations of IK/Ku80 Are Associated with Higher Tumor Mutation Burden

**Figure 5:**
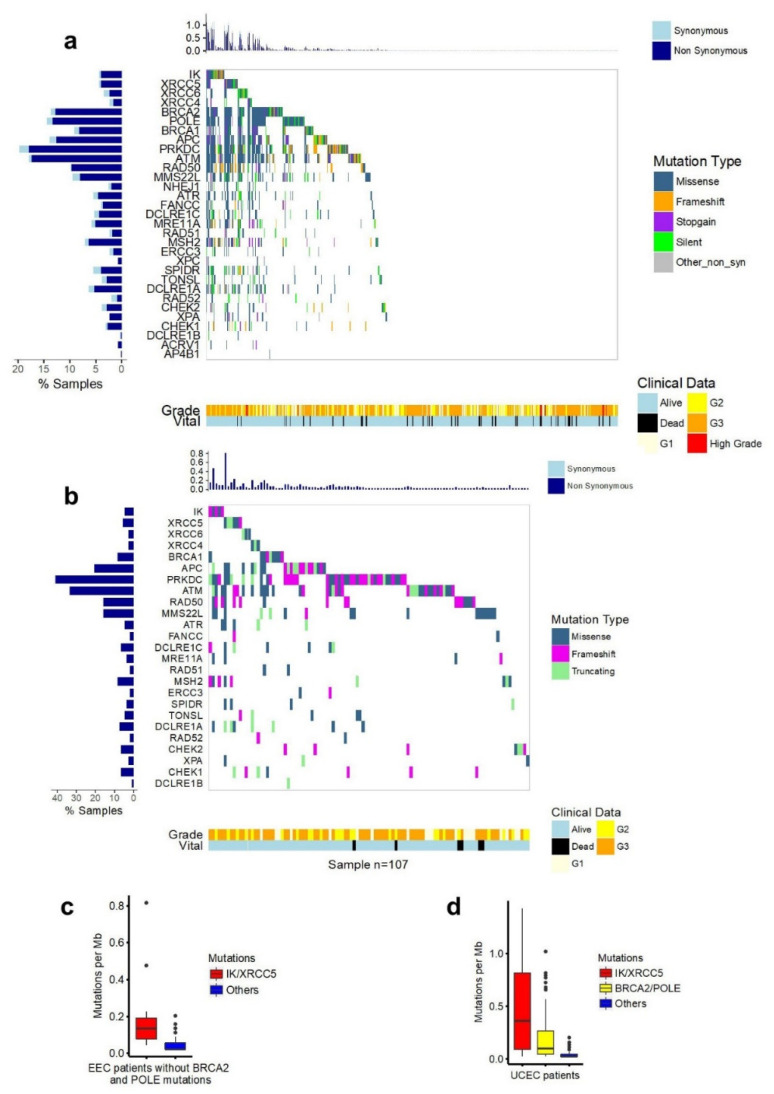
CoMut plot of DNA repair genes and mutation burden of patients with *IK/Ku80* mutations. (**a**) CoMut plot of DNA repair genes. The grade and vital status information of 547 EEC patients in TCGA were included. The somatic mutations for 3′ UTR and 5′UTR were excluded. Truncating category is for frameshift, stop-gain, stop-loss, and nonsense mutations. (**b**) coMut plot of DNA repair genes for patients without *BRCA2/POLE* mutations. (**c**) Excluding impact of mutation of *BRAC2* or *POLE* on mutation burden of other DNA repair genes, *IK* or *XRCC5(Ku80)* mutations resulted in significantly higher mutation burden than wild-type. *p* < 1.0 × 10^−7^, Mann–Whitney U test. (**d**) Patients with EEC were divided into 3 groups: *IK/XRCC5* (patients with *IK* and/or *XRCC5* mutations with or without *BRCA2/POLE* mutations); *BRCA2/POLE* (patients with mutations of *BRCA2* and/or *POLE* mutations, but not *IK/XRCC5* mutations); and all others (patients without *IK/XRCC5* or *BRCA2/POLE* mutations). *IK/XRCC5* vs. *BRCA2/POLE*, *p* < 1.0 × 10^−5^, Mann–Whitney U test; *BRCA2/POLE* vs. others, *p* <1.0 × 10^−12^, Mann–Whitney U test.

**Figure 6:**
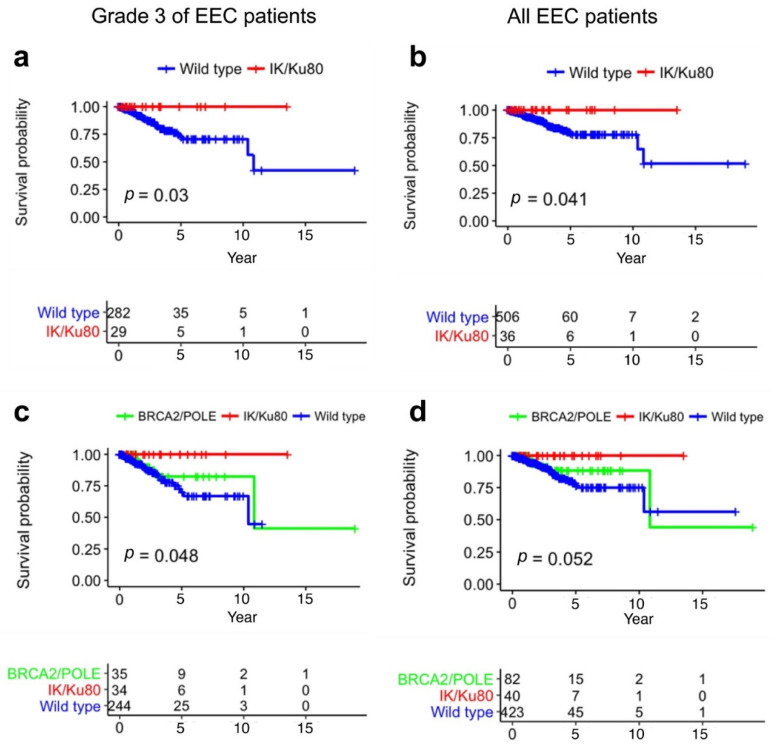
Survival analyses for EEC patients in TCGA differentiated by mutations of *IK*, *Ku80*, *BRCA2*, and *POLE*. (**a**) For EEC patients with Grade 3 disease, *IK* and *Ku80* mutations were associated with significantly better overall survival; patients with either *IK* or *Ku80* mutations were all alive. *p* = 0.03; (**b**) For all EEC patients, *IK* and *Ku80* mutations were associated with significantly better overall survival and patients with either *IK* or *Ku80* mutations were all alive. *p* = 0.041; (**c**) *BRCA2* and *POLE* mutations were associated with significantly better overall survival in *IK/Ku80* wild-type cases of Grade 3 EEC. *p* = 0.048; (**d**) *BRCA2* and *POLE* mutations were associated with better overall survival in *IK/Ku80* wild-type cases among all EEC patients, *p* = 0.052. Log-rank test.

To compare the role of *IK* to that of other genes involved in repair of DNA mutations, we analyzed co-mutation patterns of *IK* with 30 DNA repair genes in the TCGA dataset (n = 547) and presented the results in a coMut plot (Figure 5a and Table S5). The mutation rates for patients with *IK, Ku80*, and *Ku70* mutations were higher than for those without these mutations. After excluding 82 cases with either *BRCA2* or *POLE* mutations (associated with high mutation burden [28,29]), the remaining 40 cases with *IK/Ku80* mutations still had increased mutation burdens compared with the 425 wild-type cases (*p* < 1.0 × 10^−7^, Mann–Whitney *U* test) (Figure 5b–d). *IK/Ku80* mutations were associated with longer survival in EEC patients with Grade 3 disease (*p* = 0.03) or for all EEC patients (*p* = 0.041) (Figure 6a,b). Similarly, *BRCA2* and *POLE* mutations were also associated with better overall survival in *IK/Ku80* wild-type cases of EEC (Figure 6c,d).

## 3. Discussion

In this study, we expanded on our previous work showing that mutations of the *IK* gene were found primarily in Cluster IV of EEC samples within the TCGA cohort [11]. Intriguingly, patients in Cluster IV (late-stage and high-grade EEC) had longer survival than those in Cluster II. The most noteworthy mutational event in Cluster IV was *IK* gene mutation. Here, we interrogated the expanded TCGA cohort of 547 EEC cases and validated the association of *IK* mutations with better prognosis. All EEC cases with *IK* mutations were alive at 10-year follow-up in TCGA. In addition, our functional studies revealed that IK knockdown led to enrichment of G2/M phase cells, inactivation of DNA repair signaling mediated by heterodimerization of Ku80 and Ku70, and sensitization of EC cells to cisplatin treatment.

IK is known as a mitotic regulator, although its functions have not been extensively characterized. According to the bioinformatics analysis, a fraction (approximately 28% of nonsynonymous mutations) of *IK* mutations were frameshift and stop-gain, which would likely result in a loss-of-function truncated form of the protein. Our analysis also showed that the EC with these two types of mutations had decreased transcript levels compared with other EC cases. Based on these observations, loss of function is likely a key mechanism underlying *IK* mutations in EC. Loss of function via frameshift mutations has been seen with two other well-studied tumor suppressor genes, APC and BRCA1/2 [30,31]. Using siRNA knockdown technology to phenocopy the loss of function of IK, we evaluated the role of inactivating *IK* gene mutations in cell cycle. Interestingly, we observed heterogeneous impact on mitosis. IK attenuation led to enrichment of G2/M phase cells in Ishikawa cells. Similar results have been reported in other studies [19,20,32]. However, we did not find obvious enrichment of G2/M phase cells in KLE cells after IK siRNA transfection. We inferred that this was because of the long doubling time of KLE cells as poorly differentially EC cell types [33]. We found that the subG1 phase cells increased after IK siRNAs transfection in both Ishikawa and KLE cells. In addition, caspase 3/7, caspase 8, and caspase 9 were also activated. Further, when we used Q-VD-OPH, an effective cell apoptosis inhibitor [34], it decreased cell death ratio caused by IK attenuation. All of these data showed that IK attenuation caused cell apoptotic death through intrinsic mitochondria-dependent and extrinsic death receptor-dependent pathways and inhibited cell proliferation [35].

Platinum-based chemotherapy is commonly used to treat EC patients, especially patients with advanced diseases [22]. Our experiments demonstrated that IK attenuation sensitized EC cells to cisplatin treatment in vitro and in vivo. This is similar to previous reports on the association of *BRCA2* mutations with increased response to cisplatin in ovarian cancer [36]. Our in vivo studies showed that IK attenuation inhibited EC tumor growth. Mechanistically, we showed that IK interacted with Ku80 and IK attenuation negatively modulated heterodimerization between Ku80 and Ku70. In the NHEJ pathway, the Ku70/80 heterodimer forms a ring and binds to broken DNA ends directly and recruits DNA protein kinases, such as DNA-dependent protein kinase catalytic subunit (DNA-PKcs) [37,38]. Subsequently, DNA ligase IV, X-ray repair complementing defective repair in Chinese hamster cells 4 (XRCC4), and Xrcc4-like factor (XLF) are recruited and form a complex together which can rejoin the broken DNA ends [39,40,41]. Formation of the Ku heterodimer occurs upstream from the NHEJ pathway [42]. Once this heterodimer is dissociated, a single Ku80 or Ku70 unit cannot bind to broken DNA ends and then the NHEJ pathway cannot be activated. Previous in vivo studies also demonstrated that inactivation of Ku70 or Ku80 led to multiple defects, like hypersensitivity to radiation [43]. Here we showed that IK attenuation negatively modulated heterodimerization between Ku80 and Ku70. Then the ability of NHEJ decreased.

Most cancer cells, including EC cells, proliferate more rapidly than their normal counterparts [44]. Most chemotherapies, like cisplatin, cause the most DNA damage in cycling cells. In this study, we provided evidence that IK attenuation led to DNA damage response reaction. γ-H2AX is a fast and sensitive marker of DNA damage [45], which accumulates in DNA double-strand breaks (DSBs) in 1–3 min. Our data showed that IK attenuation caused more γ-H2AX foci. DSBs are generally considered to be the most toxic type of DNA damage [44].Like NHEJ, the HR signaling pathway is also an important way to repair DSBs; both of them are cell cycle-regulated [44,46]. HR mainly functions in the S and G2 phases, while NHEJ occurs predominantly in the G1 and G2 phases [47,48]. The HR and NHEJ pathways contribute to chemoresistance [49,50]. Cells with compromised DNA repair pathways are more sensitive to apoptosis caused by DNA damage [51]. We showed that IK attenuation sensitized EC to cisplatin treatment through regulating DNA repair pathways. Consistently, we found that *IK* mutations were associated with increased tumor mutation burden independent of mutations of *BRCA* and *POLE*, two DNA repair genes associated with tumor mutation burden in EC [28,29].

Whereas the knockdown approach by siRNA appropriately modeled the loss-of- function mutations of *IK*, our studies did not address missense mutations of *IK*, which did not exhibit decreased expression in our analysis. Missense mutations of *POLE* in EC disrupt the exonuclease domain of the protein [52]. However, the impact of *IK* mutations in the poorly defined RED domain in IK protein is not clear. Limitations of the current study will need to be addressed by CRISPR/cas9-mediated introduction of missense mutations into an EC cell system for functional evaluation. Functional characterization of the domains in IK in the future will also help interpret the impact of missense mutations found in *IK* mutated EC.

## 4. Materials and Methods

### 4.1. Somatic Mutation Calling for IK and DNA Repair Genes

Somatic mutation calling was implemented by MuTect1-1.1.4 (Nature Biotechnology, New York, NY, USA) [53], VarScan2-2.3.9 (Bioinformatics, Oxford, England, UK) [54] and Somatic-SNIPER [55]. Mutation loci were annotated using ANNOVAR [56] and SnpEff [57] software. Single-nucleotide variations included silent and non-silent mutations (missense, nonsense, splice-site, stop-gain, and stop-loss). Short insertions and deletions (indels) included in-frame shift, frameshift, stop-gain, and stop-loss mutations. Mutations affecting the 3′UTR, 5′UTR, intronic, and intergenic sequences were excluded. The somatic mutation loci and genes for each sample were a union from the outputs of the three pipelines for somatic mutation calling (called by at least one calling pipeline). The raw bam sequencing data of DNA repair genes in 547 EEC samples were downloaded from the whole exome sequencing dataset at Genomics Data Commons (https://www.mdpi.com/2072-6694/13/10/2487 (accessed on 4 April 2017) using bamslicer. Mutation loci for *IK* and all DNA repair genes were visualized using OncoPrint in cBioPortal (http://www.cbioportal.org/ (accessed on 4 April 2017) and the waterfalls in the R package of GenVisR [58]. Gene expression data of IK from RNA-seq and clinical information of EEC patients were downloaded from the Genomic Data Commons. Tumor mutation burden was calculated as the number of non-synonymous somatic mutations per mega-base in coding regions.

### 4.2. Manual Inspection of an Indel of IK, exon5:c_267_267delines-GA

We specifically addressed an emerging mutation calling inconsistency regarding the indel of *IK*, exon5:c_267_267delines-GA, identified by VarScan2. This frameshift was present in the analysis of original 240 ECs [59] but not reported in the 509 ECs from the Pan-Cancer Atlas study in 2018 [60], which used a computational artifact filtering algorithm to reduce provisional mutation calls of false positives and negatives [61]. One advantage for the computational artifact filtering algorithm is the avoidance of manual inspection of binary alignment map (BAM) files, which is only feasible for a small number of samples because of the time-consuming nature of manual inspection. However, there are caveats for the computationally identified artifacts if the filtering algorithms are overly stringent: the filtered artifacts may include real mutations. This is due to the fact that the reported accuracy of the developed algorithms is about 0.90 [61].

For *IK* mutation calls, we used manual inspection for validating the indel: exon5:c_267_267delines-GA. We examined the raw sequencing data from both tumor and normal samples to determine if the initially observed indel was a false-positive. We visualized BAM files using IGV software (https://software.broadinstitute.org/software/igv/ (accessed on 3 February 2021) and thus manually inspected the c_267_267delines-GA indel in the 6 EC samples by visualizing both tumor and normal bam files. This manual inspection confirmed the existence of significant numbers of reads in the tumor BAM files, supportive of the initially identified indel (example shown in Figure S9).

We next examined if the indel was present as a germline mutation in the other 541 EC samples. We did not identify such mutations in the EC normal bam samples by VarScan2 and GATK calling pipelines c_267_267delines-GA.

Subsequently, we examined whether the repetitive sequence (of GA) adjacent to c_267_267delines-GA can cause alignment orientation error. As shown in IGV software (Figure S10), the length of reads in the alignments is much longer than that of the GA repetition. The alignments used the sequence information of both GA repetitive and other reference sequences and thus avoided the issue of artifacts from alignment orientation.

Lastly, reads from single strand can contribute to mutation call errors [62]. Analysis of read orientation on both forward and reverse strands for paired-end reads can estimate the likelihood of artifacts. In the 6 EC samples with c_267_267delines-GA, the mutated reads were revealed by both strands and did not have such strand or orientation biases (or called artifacts) (Table S6).

### 4.3. Cell Culture, Transfections, and Reagents

Three kinds of EEC cell lines (Ishikawa, KLE, and HEC1A) were obtained from the American Tissue Culture Collection (Manassas, VA, USA). Ishikawa and KLE cell lines were cultured in Dulbecco’s modified Eagle’s medium (DMEM)/F12 50:50 mix. HEC1A cell lines were cultured in McCoy’s 5A medium. The medium was supplemented with 10% fetal bovine serum and 5% penicillin/streptomycin. All cell lines were cultured in an incubator with 5% CO_2_ at 37 °C.

To attenuate IK protein expression, two different pools of small interfering RNA (siRNA) (no. 7260 and no. 7261, Thermo Fisher Scientific Inc., Waltham, MA, USA) were transfected into cells via Lipofectamine RNAiMAX (Life Technologies, Grand Island, NY, USA) according to the manufacturer’s protocol. Negative control siRNA from Thermo Fisher Scientific Inc. (no. 4390843) was also transfected into cells via Lipofectamine RNAiMAX according to the manufacturer’s protocol.

Anti-IK antibodies were purchased from Thermo Fisher Scientific Inc. (no. PA5-32098) and Santa Cruz Biotechnology (no. sc135485). The anti β-actin antibody (no. sc47778) and secondary antibodies (no. sc2302 and sc2313) were purchased from Santa Cruz Biotechnology, Inc. The anti-phosphorylated H2AX (γ-H2AX) antibody (no. 9718s) and anti-Ku80 antibody (no. 2180s) were all purchased from Cell Signaling Technology. The anti-Ku70 antibody (no. ab92450) was purchased from Abcam.

### 4.4. Western Blotting and IP Analysis

For Western blotting, whole proteins were extracted from incubating cell pellets in RIPA buffer with a 0.1% phosphatase inhibitor cocktail and purified by centrifugation. For IP, cells were subjected to lysis in IP buffer (NaCl 125 mM, HEPES 25 mM, EDTA 2.5 mM, and 0.1% NP-40). After preclearing with protein A/G agarose beads for 1 h on ice, lysates were immunoprecipitated overnight on a shaker at 4 °C with protein A/G agarose beads using antibodies to IK. Beads were washed with IP buffer and then boiled in Laemmli buffer. Proteins extracted from the cell lysates and from IP experiments were analyzed by SDS-PAGE (10%) and then transferred onto a polyvinylidene fluoride membrane (Millipore, Billerica, MA, USA). Membranes were blocked with 5% non-fat milk for 2 h at room temperature and incubated overnight at 4 °C with primary antibodies. After washing in Tris-HCl buffered saline solution with 0.1% Tween-20, blots were incubated for 1 h in 5% non-fat milk with secondary antibodies coupled to horseradish peroxidase at room temperature. Then immunoblots were incubated with SuperSignal^™^ chemiluminescent substrate (Pierce Biotechnology, Waltham, MA, USA) and developed.

### 4.5. Cell Cycle Assays

Twenty-four, 48, and 72 h after IK siRNA and negative control siRNA transfection, all cells were harvested and fixed with 70% ethanol. Then a solution containing 50 mg/mL propidium iodide (PI) and 100 mg/mL RNase I in phosphate-buffered saline (PBS) was used to stain cells. After incubating at 37 °C for 30 min, a FACScan flow cytometer with Cell Quest software (BD Biosciences, San Jose, CA, USA) was used to analyze the data.

### 4.6. Cell Viability Assays

The effects of IK siRNA on cell viability were examined with a Cell Counting Kit-8 (CCK-8) assays (Dojindo Medical Technologies, Inc., Rockville, MD, USA). Twenty-four hours after IK siRNA and negative control siRNA transfection, 4 × 10^3^ transfected Ishikawa and KLE cells in 100 μL completed medium were seeded in 96-well plates. After 24 h, 48 h, 72 h, and 96 h incubation, 10 μL CCK-8 was added to each well and cells were incubated for another 2 h. OD values were measured using a plate reader at 450 nm. CCK-8 assays were also used to detect the interaction of IK siRNA and cisplatin on cell viability. After transfected cells were seeded in 96-well plates and attached, 4 μM and 2 μM cisplatin were added to Ishikawa and KLE cells, respectively. After 24 h, 48 h, 72 h, and 96 h incubation, 10 μL CCK-8 was added to each well and cells were incubated for another 2 h. OD values were measured using a plate reader at 450 nm.

### 4.7. Colony Formation Assays

Colony formation assays were used to detect effects of IK siRNA on cell proliferation, as previously described [63]. Briefly, negative control siRNA-treated and IK siRNA-treated Ishikawa and KLE cells were placed into 6-well coated high-adhesion plates. They were cultured for 7 days. Then plates were washed with PBS twice. After washing, 1 mL methanol containing crystal violet solution (0.025% *w*/*v*; Sigma) was used to fix and stain cells for 5 min. The number of colonies was counted in each well.

### 4.8. Cell Death Assays

We examined effects of IK siRNA and cisplatin on cell death using trypan blue exclusion assays. Twenty-four hours after IK siRNA and negative control siRNA transfection, 4 μM and 2 μM cisplatin were added to Ishikawa and KLE cells, respectively. After another 48 h of incubation, both floating and adherent cells were harvested and resuspended in 1 mL PBS. Cell death ratio was calculated as the percentage of positive stained cells (blue)/ total cells.

### 4.9. Cell Apoptosis Assays

We examined effects of IK siRNA and cisplatin on cell apoptosis with the annexin V-FITC/PI Apoptosis Detection Kit (BD Biosciences) according to the manufacturer’s instructions. Twenty-four hours after IK siRNA and negative control siRNA transfection, 4 μM and 2 μM cisplatin were added to Ishikawa and KLE cells, respectively. After another 48 h of incubation, both floating and adherent cells were harvested and suspended in a binding buffer provided with the kit. Then, 5 µL of annexin V-FITC and 5 µL of PI were used to stain cells. After incubation in the dark for 20 min at room temperature, a FACScan flow cytometer with Cell Quest software (BD Biosciences) was used to analyze the data.

### 4.10. Tumor Growth Assays

Female athymic nude mice were purchased from Taconic Biosciences (Rensselaer, NY, USA) and maintained according to guidelines set forth by the American Association for Accreditation of Laboratory Animal Care and the US Public Health Service Policy on Human Care and Use of Laboratory Animals. All mouse studies were approved by the M.D. Anderson Cancer Center Institutional Animal Care and Use Committee. Mice used in this study were between 8 and 12 weeks of age at the time of injection.

For cell injection, Ishikawa or HEC1A cells were trypsinized, washed, and suspended in Hanks’ balanced salt solution (Gibco, Carlsbad, CA, USA) before injection. To establish tumors, 100 µL cells (4.0 × 10^6^ for Ishikawa or 1.0 × 10^6^ for HEC1A) in 20% Matrigel were injected subcutaneously over the right flank of each mouse. Mice were randomly and equally assigned to four groups (control siRNA-DOPC, control siRNA-DOPC plus cisplatin, IK siRNA-DOPC, and IK siRNA-DOPC plus cisplatin). The siRNAs for these experiments were incorporated into DOPC nanoliposomes, as described previously [64,65]. All treatments were initiated five days after injection.

Cisplatin (160 µg/mouse or 6.4 mg/kg) was given intraperitoneally once weekly after being dissolved in PBS. A dose of 200 µg/kg of either control siRNA-DOPC or IK siRNA-DOPC was delivered intraperitoneally twice weekly. Mice were monitored daily and tumor volume was measured twice weekly. Mice were sacrificed when they became moribund (approximately four weeks after injection of cells), and their body weights, tumor weights, and tumor volumes were recorded.

### 4.11. Silver Staining Assays

Proteins from IP experiments were analyzed on SDS-PAGE (7%) columns and the resulting gels were stained with a Silver Quest Staining Kit (Thermo Fisher Scientific Inc., Waltham, MA, USA) according to the manufacturer’s instructions. Stained gels were cut into several small bands following the differences between the IgG control and IK antibody pull down lanes. Then these bands were used for further mass spectrographic analysis.

### 4.12. DNA Damage/γ-H2AX Assays

Twenty-four hours after IK siRNA and negative control siRNA transfection, 5 × 10^3^ Ishikawa and KLE cells per well were seeded in 96-well plates. After another 48 h of incubation, DNA damage was detected using EpiQuik In Situ DNA Damage Assay kits (EpiGentec, Farmingdale, NY, USA) according to the manufacturer’s instructions [66]. For γ-H2AX assays, 1 × 10^5^ cells were seeded in chamber slides and incubated for another 48 h at 37 °C. Then the cells were fixed in 4% paraformaldehyde and blocked with 5% goat serum. After that, the cells were incubated with a primary antibody to γ-H2AX (1:400) at 4 °C overnight. They were then incubated with a secondary antibody (Alexa Fluor 488 goat anti-rabbit IgG, 1:800) for 1 h at room temperature. Nuclei were counterstained with 4′, 6-diamidino-2-phenylindole, dihydrochloride (DAPI). Then an Olympus FV1000 laser confocal microscope was used to acquire immunofluorescence images at a × 40/NA 1.3 objective.

### 4.13. DNA Repair Assays

Efficiency of HR was measured using a quantitative polymerase chain reaction (qPCR)-based assay kit (Norgen Biotek Corp., Thorold, ON, CA) [67,68]. Briefly, this kit consists of two pUC19 plasmids (dl1 and dl2); each plasmid has a different mutation in its lacZ coding region. Twenty-four hours after IK siRNA transfection, these two plasmids were co-transfected (2.5 μg of dl1 and 2.5 μg of dl2 per six-well plate) and cells were incubated for another 48 h. Then total cellular DNA was isolated using a Genomic DNA Mini Kit (Thermo Fisher Scientific Inc.). Either a set of universal primers amplifying all plasmid backbones or a set of primers that only amplify plasmid DNA generated by HR was used for qPCR with isolated cellular DNA. PCR reactions were performed under the following conditions: 95 °C for 3 min, followed by 40 cycles of 95 °C for 15 s, 61 °C for 30 s, and 72 °C for 1 min.

NHEJ assays were done as described by Kristen et al. [69]. Briefly, 72 h after IK siRNA transfection, nuclear protein was extracted using a PER/NER fractionation kit (Pierce). pBlueScriptII SK^+^ plasmid was linearized using SacI-HF (New England Biolabs, Ipswich, MA, USA). The linearized plasmid was resolved on a 0.7% agarose gel and gel-extracted via the Wizard SV Gel Cleanup System (Promega, Madison, WI, USA). Then 2 μg of nuclear protein and 100 ng of the linearized plasmid were incubated in end joining buffer (1 mM ATP, 100 mM potassium acetate, 25 mM Tris acetate, 0.25 mM dNTP, 1 mM DTT, 10 mM magnesium acetate). The mixture was incubated for 1 h at 37 °C in a water bath, followed by protein digestion with proteinase K for 30 min at 65 °C in a water bath. Then the mixture was diluted 1:1000 and 4 μL of the diluted mixture was used in two qPCR assays: internal control and NHEJ repair. The pBlueScript primers T7 and Sk were used to generate an internal control amplicon and T7 and T3 were used to generate the repair amplicon. The total qPCR reaction volume was 15 μL under the following conditions: 95 °C for 10 min, then followed by 40 cycles of 95 °C for 30 s and 60 °C for 60 s. The cycle number and ΔΔ Ct methods were used to evaluate the amount of HR and NHEJ products for each reaction.

### 4.14. Statistical Analysis

Standard statistical tests were used to analyze clinical and genomic data, including the Mann-Whitney U, Chi-squared, Fisher’s exact, Student *t*, and log-rank tests. Cox proportional hazards analysis was used to obtain statistical significance for associations between mutation and survival. Analyses were primarily performed using the R package (https://www.r-project.org/ (accessed on 4 April 2017–1 July 2017). Survival analyses were implemented in the “Survival” and “KMsurv” R packages. Statistical significance was defined as a *p* value less than 0.05. All statistical tests were two-sided. For in vitro experiments, all data were expressed as the mean ± standard deviation (SD) of at least 3 independent experiments. The two-sided Student’s *t* test was used to compare differences between two independent groups. Statistical significance was defined as a *p* value less than 0.05. GraphPad Prism 7 (GraphPad Software, San Diego, CA, USA) and SPSS 22.0 (IBM, Armonk, NY, USA) software were used for statistical analysis.

## 5. Conclusions

Taken together, our integrated omics and functional studies characterized an unrecognized function of the *IK* gene in DNA repair. Inactivating mutations of *IK* in EC improved response to chemotherapy, resulting in longer survival. This study provides a foundation to further develop IK as a prognosticator. Future studies will determine whether *IK* mutations can contribute to new molecular classification of EC and how *IK* mutations affect prognosis in a broader spectrum of cancers.

## Data Availability

The data presented in this study are openly available at https://www.cancer.gov/about-nci/organization/ccg/research/structural-genomics/tcga, http://software.broadinstitute.org/software/igv/, https://www.mdpi.com/2072-6694/13/10/2487, http://www.cbioportal.org/, https://software.broadinstitute.org/software/igv/ and https://www.r-project.org.

## Supplementary Materials

The following are available online at www.mdpi.com/user/manuscripts/displayFile/30c29744134be920b54b545f16e78fac/supplementary. Table S1: Clinical parameters of 547 EEC patients in TCGA dataset, Table S2: The somatic mutations for *IK*, Table S3: Distribution of EEC patients with *IK* mutations, Table S4: Vital status of *IK* mutated EEC patients and wild-type cases, Table S5: DNA repair genes, Table S6: Reads discovered c_267_267delines-GA indel are not from a single strand, Figure S1: IK attenuation affects cell cycle in Ishikawa and KLE cells, Figure S2: IK attenuation causes cell apoptosis in Ishikawa and KLE cells, Figure S3: IK attenuation causes apoptotic cell death through intrinsic mitochondria dependent and extrinsic death receptor dependent pathways in Ishikawa and KLE cells, Figure S4: IK attenuation inhibits cell viability and cell proliferation in Ishikawa and KLE cells, Figure S5: IK attenuation sensitizes EC to cisplatin in KLE cells, Figure S6: IK attenuation inhibits EC cell growth and sensitizes EC to cisplatin in vivo, Figure S7: Mass spectrometry result showed that IK interacted with Ku80, Figure S8: IK attenuation leads to inactivation of DNA repair signaling in KLE cells, Figure S9: Example of manual inspection to confirm the existence of significant numbers of reads in the tumor BAM files, supportive of the initially identified indel, Figure S10: IGV software showed that the length of reads in the alignments is much longer than that of the GA repetition.
