# Supplementary material for: Inactivating Mutations of the IK Gene Weaken Ku80/Ku70-Mediated DNA Repair and Sensitize Endometrial Cancer to Chemotherapy
Source: Cancers (Basel). 2021 May 20;13(10):2487. doi: 10.3390/cancers13102487 (PMC8160817; doi:10.3390/cancers13102487)
Supplement: Supplementary file 1 [file cancers-13-02487-s001.zip › cancers-1188938-supplementary-final/Supplementary material.pdf]

Table S1. Clinical parameters of 547 EEC patients in TCGA dataset.

Clinical Parameters	Name	Statistics
**Primary Site**	Corpus uteri	547 (100%)
**Gender**	Female	547 (100%)
**Vital Status**	Alive	456 (83.3%)
	Dead	91 (16.7%)
**Race**	White	374 (68.2%)
	Black or African American	100 (18.2%)
	Asian	20 (3.6%)
	Not reported	31 (5.8%)
	Other	13 (2.3%)
**Ethnicity**	Not hispanic or latino	376 (68.8%)
	Hispanic or latino	15 (2.7%)
	Not reported	156 (28.5%)
**Stage**	Stage I, IA, IB, IC	341 (62.4%)
	Stage II, IIA, IIB	52 (9.5%)
	Stage III, IIIA, IIIB, IIIC, IIIC1, IIIC2	122 (22.3%)
	Stage IV, IVA, IVB	32 (5.8%)

Table S3. Distribution of EEC patients with *IK* mutations.

Group	G1	G2	G3	High-grade
All patients	99	121	313	11
With *IK* mutations	3	4	30	0
synonymous SNV	0	0	5	
stopgain	0	0	3	
nonsynonymous SNV	0	2	18	
frameshift substitution	0	2	3	

* p -value < 2.2e-16, Fisher's exact test.

Table S4. Vital status of *IK* mutated EEC patients and wild-type cases.

Group	Death	Alive
All patients	44 (0.080)	503 (0.919)
With mutations	0	32
No mutations	44	471

* p -value < 0.05.

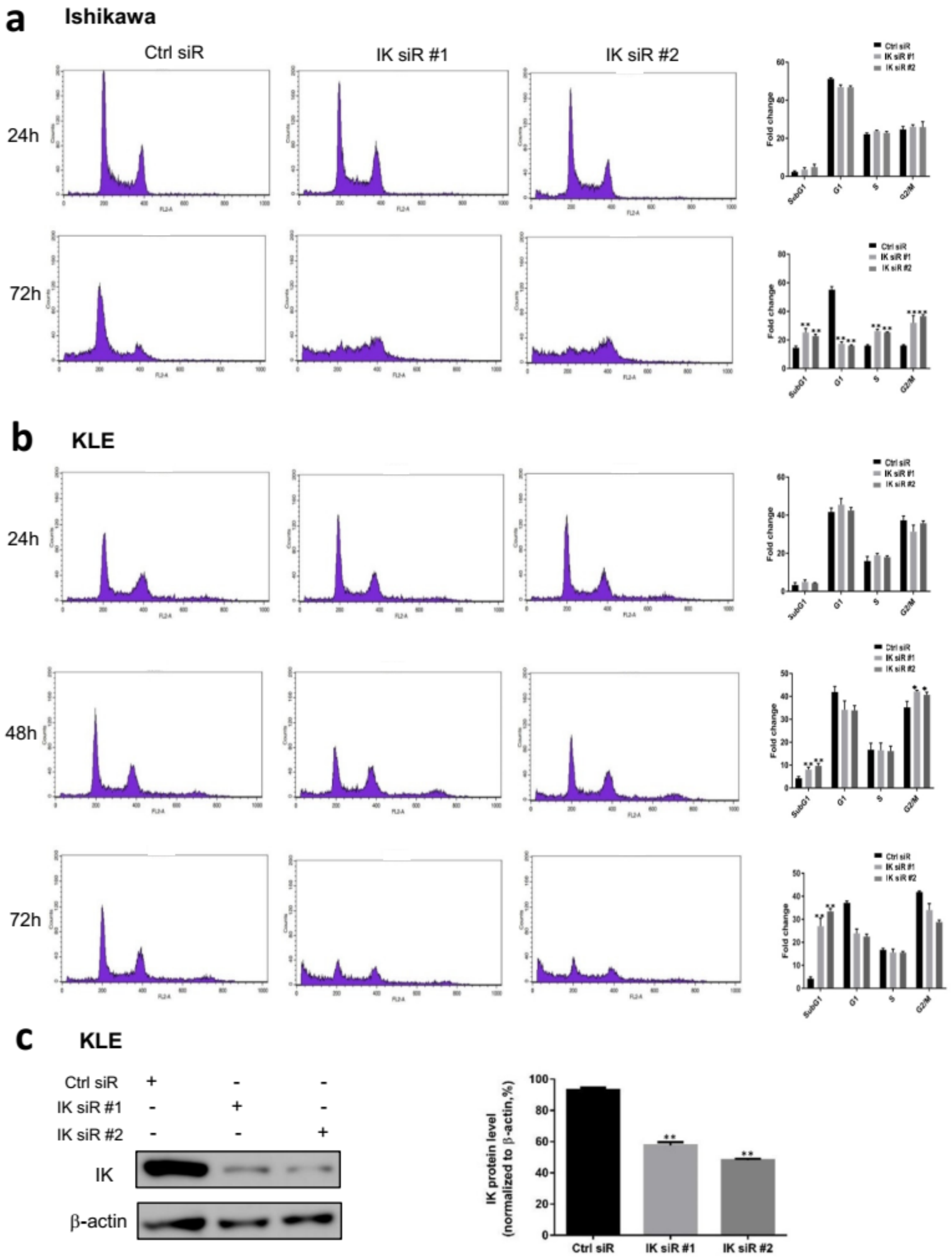

Figure S1. IK attenuation affects cell cycle in Ishikawa and KLE cells. **(a)**. (Left) Twenty-four and seventy-two hours after IK siRNA transfection in Ishikawa cells, IK attenuation led to enrichment of G2/M cells. (Right) Quantification of Ishikawa cells in different phases. (**b**). (Left) Twenty-four, forty-eight and seventy-two hours after IK siRNA transfection in KLE cells, IK attenuation affected cell cycle. (Right) Quantification of KLE cells in different phases. **c**. (Left) Seventy-two hours after IK siRNA transfection in KLE cells, IK expression was attenuated. (Right) Quantitative analysis of IK protein expression. Mean ± SD of at least three independent experiments. (two-sided Student's *t* test, * p <0.05, ** p <0.01, *** p <0.001).

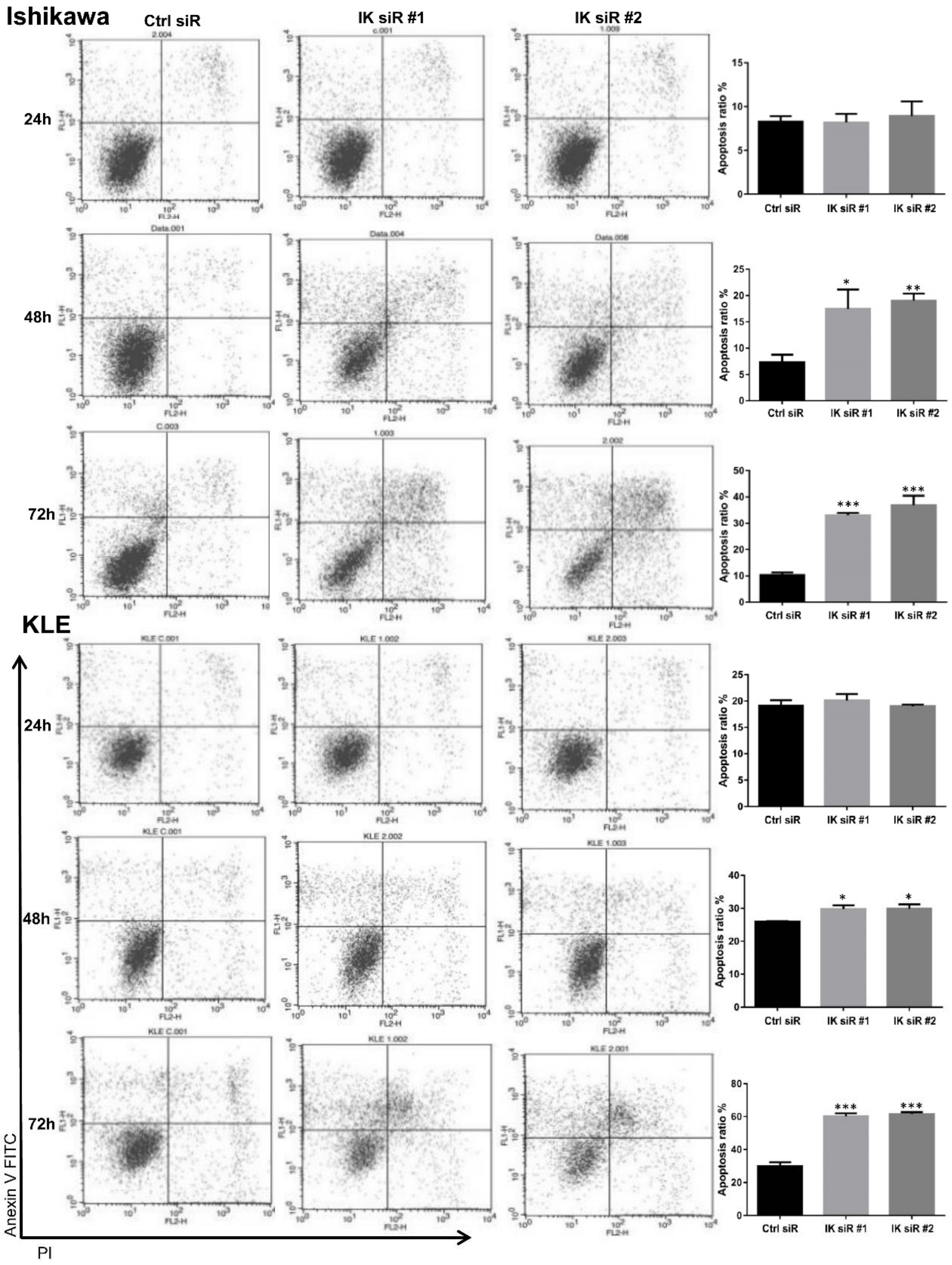

Figure S2. IK attenuation causes cell apoptosis in Ishikawa and KLE cells**.** Different times after IK siRNA transfection, all the cells, including attached and floating cells, were harvested and stained with annexin V-FITC and PI. Then they were analyzed by flow cytometry for cell apoptosis. Mean ± SD of at least three independent experiments. (two-sided Student's *t* test, * p <0.05, ** p <0.01, *** p <0.001).

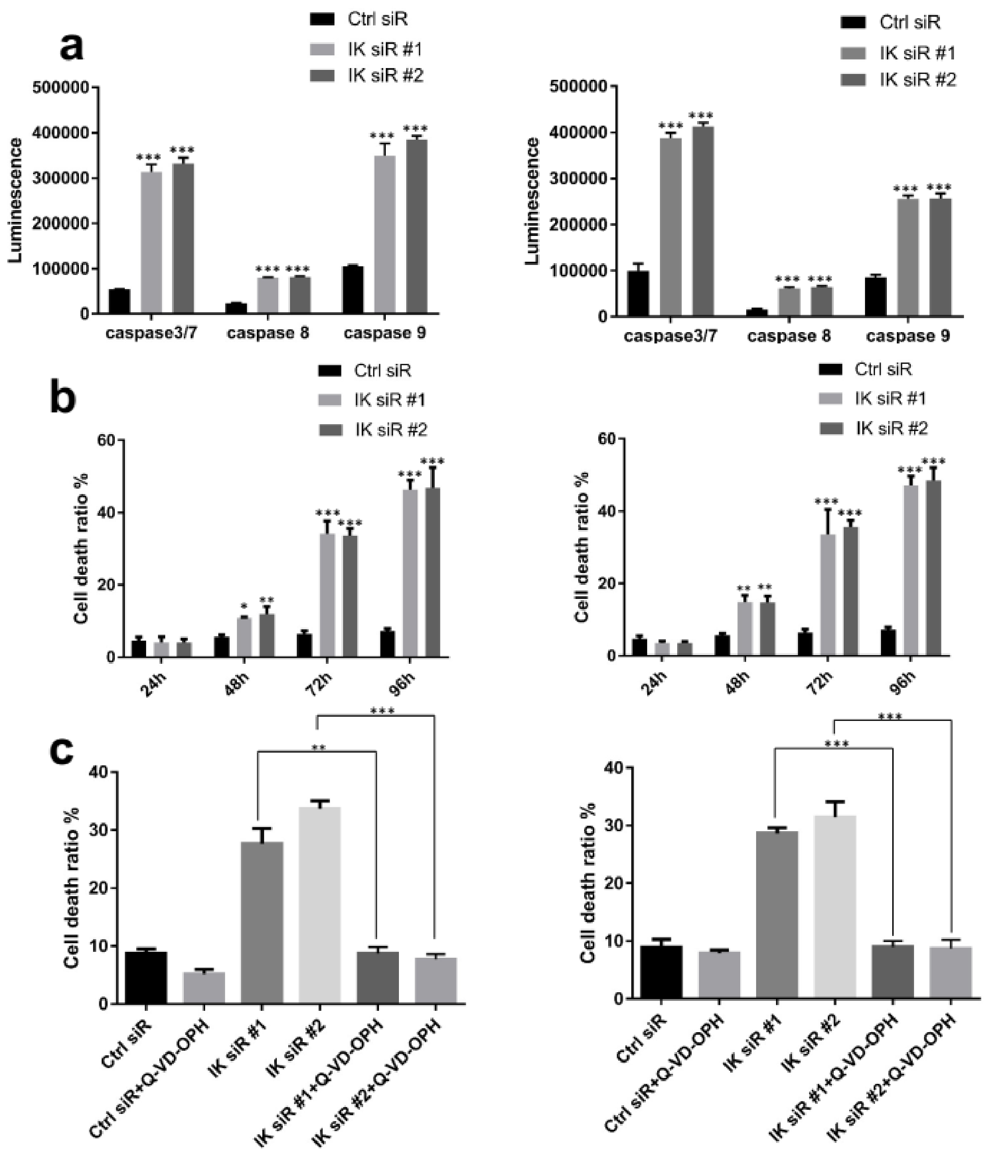

Figure S3. IK attenuation causes apoptotic cell death through intrinsic mitochondria dependent and extrinsic death receptor dependent pathways in Ishikawa and KLE cells. (**a**). Seventy-two hours after IK siRNA transfection in Ishikawa (left) and KLE (right) cells, caspase activity assay showed that caspase3/7, caspase 8 and caspase 9 were activated. (**b**). Trypan blue exclusion assay showed that IK attenuation caused cell death. c. Seventy-two hours after IK siRNA transfection with or without Q-VD-OPH (cell apoptosis inhibitor) treatment, trypan blue exclusion assay showed that Q-VD-OPH decreased cell death ratio caused by IK attenuation. Mean ± SD of at least three independent experiments. (two-sided Student's *t* test, * $p < 0.05$, ** $p < 0.01$, *** $p < 0.001$).

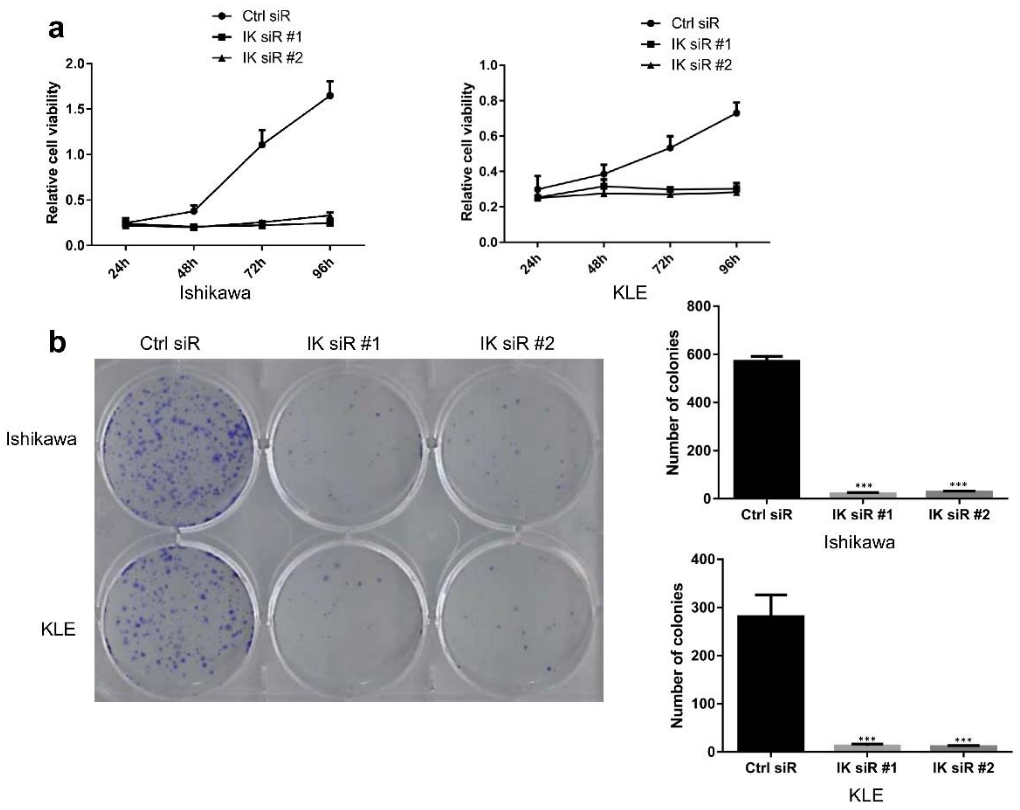

Figure S4. IK attenuation inhibits cell viability and cell proliferation in Ishikawa and KLE cells. **(a)**. After IK siRNA transfection in Ishikawa and KLE cells, cell viability was inhibited significantly. (**b**). (Left) Transfected cells were seeded in a 6 well plate (700 cells/well) and incubated for 2 weeks. Then cells were stained by 0.1% crystal violet. (Right) Quantification of colonies in Ishikawa and KLE cells. Mean ± SD of at least three independent experiments. (two-sided Student's *t* test, *** $p < 0.001$).

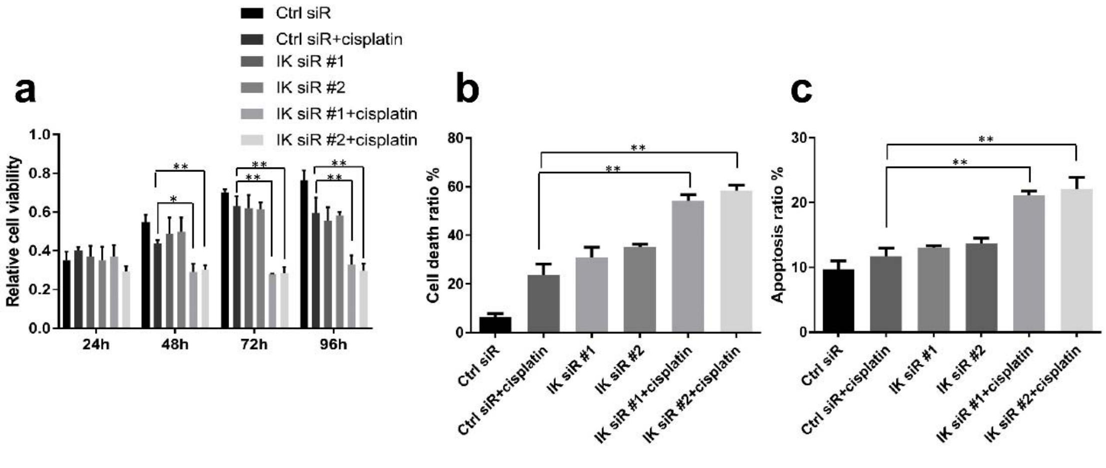

Figure S5. IK attenuation sensitizes EC to cisplatin in KLE cells. (**a**). CCK-8 assay showed IK attenuation sensitized KLE cells to cisplatin treatment. The IK siRNA transfection plus cisplatin group inhibited cell viability more significantly. (**b**). Seventy-two hours after IK siRNA transfection with or without cisplatin treatment, the IK siRNA transfection plus cisplatin group had more dead cells on trypan blue exclusion assays. (**c**). Seventy-two hours after IK siRNA transfection with or without cisplatin treatment, the IK siRNA transfection plus cisplatin group had more apoptotic cells. Mean ± SD of at least three independent experiments. (two-sided Student's *t* test, * $p < 0.05$, ** $p < 0.01$).

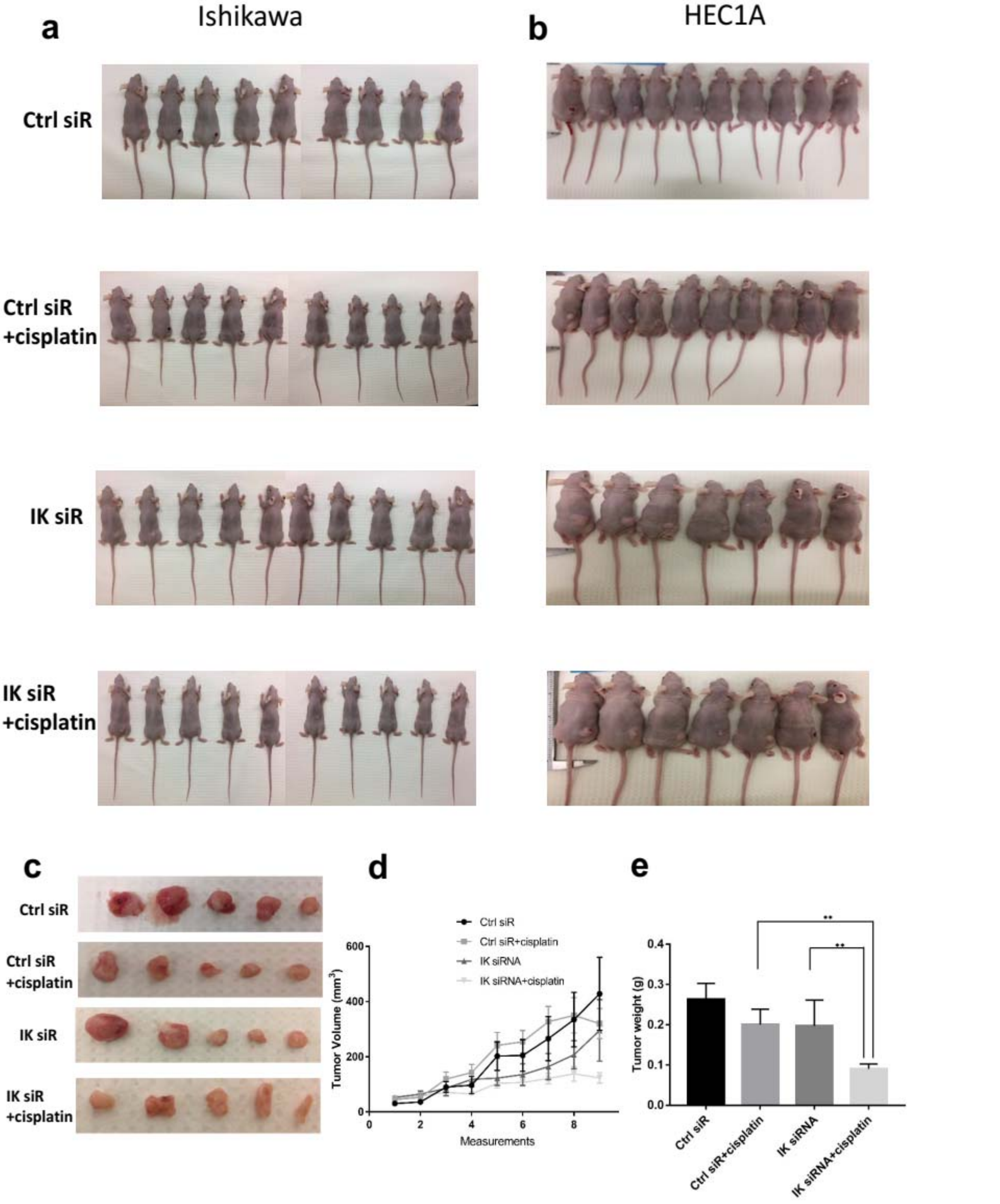

Figure S6. IK attenuation inhibits EC cell growth and sensitizes EC to cisplatin *in vivo*. (**a**). Images of Ishikawa xenograft model. (**b**). Images of HEC1A xenograft model. **c**. Representative images of HEC1A xenograft tumors in nude mice treated with control siRNA-DOPC, control siRNA-DOPC plus cisplatin, IK siRNA-DOPC, or IK siRNA-DOPC plus cisplatin (n=10 per group). Tumor volume (**d**) and tumor weight (**e**) of HEC1A xenograft tumors in each group 4 weeks after different treatments. (two-sided Student's *t* test, * p <0.05, ** p <0.01, *** p <0.001).

Probability Legend: over 95% | 80% to 94% | 50% to 79% | 20% to 49% | 0% to 19%

#	Visible?	Starred?	MS/MS View: 66 Proteins in 45 Clusters With 33 Hidden	Accession Number	Molecular Weight	Protein Grouping Ambiguity	2liz: 161218_13_2liz-1.raw (F007516...	2liz: 161218_17_2liz-2.raw (F007515...	2liz: 161218_21_2liz-3.raw (F007514...	2liz: 161218_25_2liz-4.raw (F007513...	2liz: 161218_29_2liz-5.raw (F007512...
1			ARF GTPase-activating protein GIT1 isoform 1 [Homo sapiens]	NP_001078923.1 (+1)	85 kDa	★				15	2
2			ARF GTPase-activating protein GIT2 isoform 1 [Homo sapiens]	NP_476510.1 (+1)	85 kDa	★				9	2
3			catenin beta-1 [Homo sapiens]	NP_001091679.1 (+8)	85 kDa	★				8	
4			granulins precursor [Homo sapiens]	NP_002078.1 (+1)	64 kDa					4	16
5			Cluster of vimentin [Homo sapiens] (NP_003371.2)	NP_003371.2 [4]	54 kDa	★	3	2		3	2
6			nuclear pore complex protein Nup88 isoform 1 [Homo sapiens]	NP_001307582.1	86 kDa					2	
7			transferrin receptor protein 1 isoform 1 [Homo sapiens]	NP_001121620.1 (+2)	85 kDa					2	
8			Cluster of 78 kDa glucose-regulated protein precursor [Homo sapiens] (NP_005338.1)	NP_005338.1 [4]	72 kDa	★					31
9			rho guanine nucleotide exchange factor 7 isoform a [Homo sapiens]	NP_001106985.1 (+15)	73 kDa						17
10			RNA-binding protein EWS isoform 3 [Homo sapiens]	NP_001156757.1 (+13)	68 kDa						7
11			nucleolin [Homo sapiens]	NP_005372.2	77 kDa				3		5
12			protein Red [Homo sapiens]	NP_006074.2	66 kDa						4
13			far upstream element-binding protein 2 [Homo sapiens]	NP_003676.2 (+1)	73 kDa						4
14			stress-70 protein, mitochondrial precursor [Homo sapiens]	NP_004125.3	74 kDa						2
15			X-ray repair cross-complementing protein 5 [Homo sapiens]	NP_066964.1	83 kDa						2
16			tight junction protein ZO-2 isoform 3 [Homo sapiens]	NP_001163887.1 (+7)	137 kDa		16				
17			Cluster of actin, cytoplasmic 1 [Homo sapiens] (NP_001092.1)	NP_001092.1 [9]	42 kDa	★	3	2	2		
18			Cluster of hemoglobin subunit beta [Homo sapiens] (NP_000509.1)	NP_000509.1 [2]	16 kDa	★	2		2		
19			heterogeneous nuclear ribonucleoprotein U-like protein 2 [Homo sapiens]	NP_001073027.1	85 kDa		2				
20			heterogeneous nuclear ribonucleoprotein U isoform b [Homo sapiens]	NP_004492.2 (+4)	89 kDa			4			
21			Cluster of cadherin-1 isoform 1 preproprotein [Homo sapiens] (NP_004351.1)	NP_004351.1 [3]	97 kDa	★		3			
22			glyceraldehyde-3-phosphate dehydrogenase isoform 1 [Homo sapiens]	NP_001276674.1 (+2)	36 kDa			3			
23			cystatin-A [Homo sapiens]	NP_005204.1	11 kDa			2			
24			arginase-1 isoform 2 [Homo sapiens]	NP_000036.2 (+2)	35 kDa			2			
25			sodium/potassium-transporting ATPase subunit alpha-1 isoform a [Homo sapiens]	NP_000692.2 (+4)	113 kDa			2			
26			catenin alpha-1 isoform 2 [Homo sapiens]	NP_001277236.1 (+8)	93 kDa				10		
27			elongation factor 2 [Homo sapiens]	NP_001952.1	95 kDa				2		

Figure S7. Mass spectrometry result showed that IK interacted with Ku80.

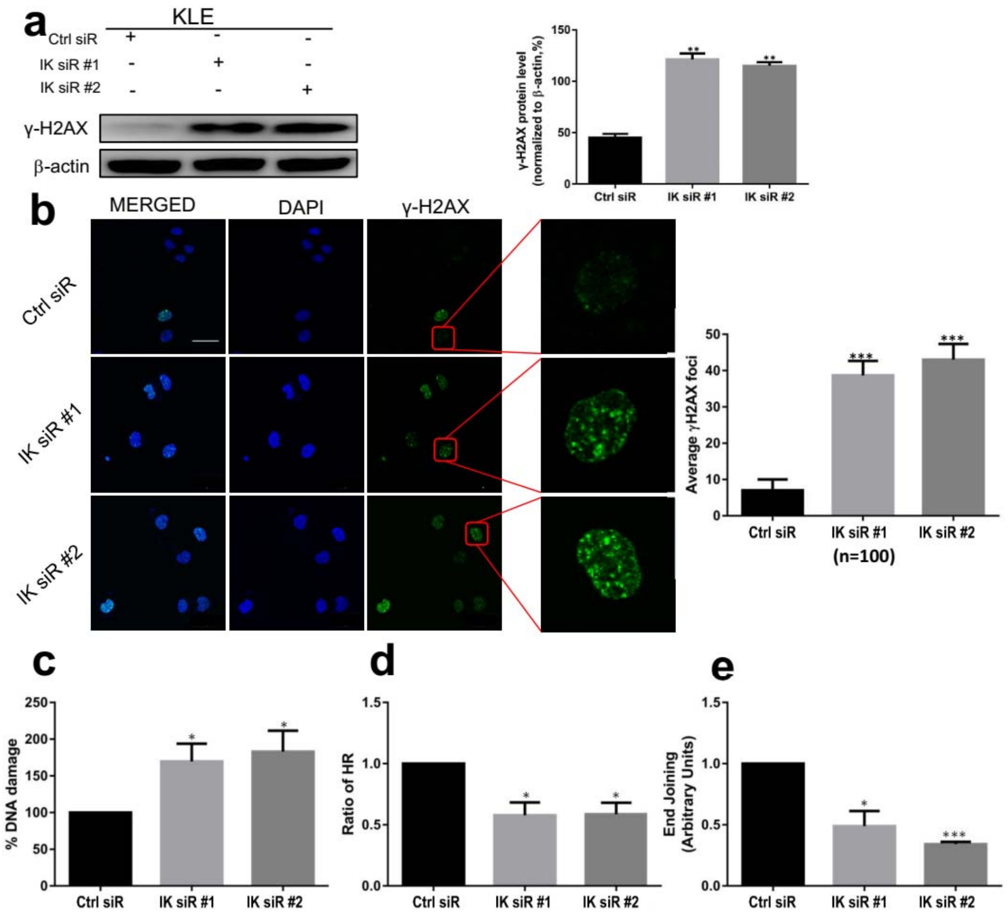

Figure S8. IK attenuation leads to inactivation of DNA repair signaling in KLE cells. **(a)**. (Left) Seventy-two hours after IK siRNA transfection in KLE cells, γ-H2AX expression increased. (Right) Quantitative analysis of γ-H2AX protein expression. (**b**). (Left) Seventy-two hours after IK siRNA transfection, IK attenuation caused more γ-H2AX foci. (Right) Quantification of average γH2AX foci per cell. (**c**). Seventy-two hours after IK siRNA transfection, IK attenuation caused more DNA damage. (d). Seventy-two hours after IK siRNA transfection, IK attenuation weakened HR efficiency. e. Seventy-two hours after IK siRNA transfection, we measured end joining in Ishikawa nuclear extracts of different groups with

qPCR; IK attenuation weakened NHEJ efficiency. Mean ± SD of at least three independent experiments. (two-sided Student's t test, * p <0.05, ** p <0.01, *** p <0.001).

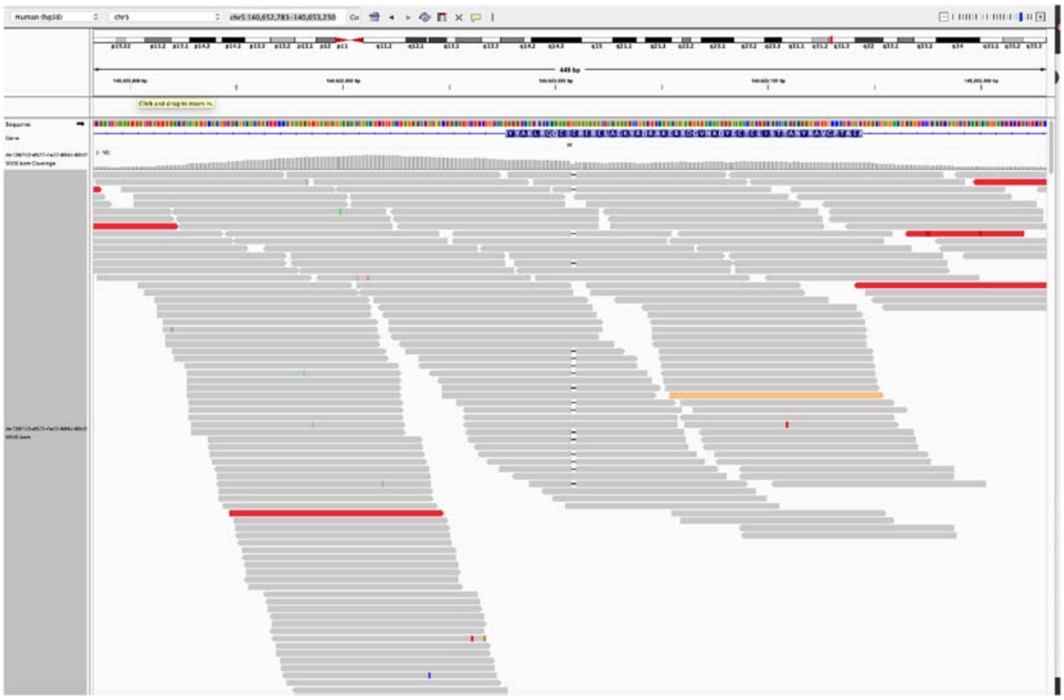

Figure S9. Example of manual inspection to confirm the existence of significant numbers of reads in the tumor BAM files, supportive of the initially identified indel.

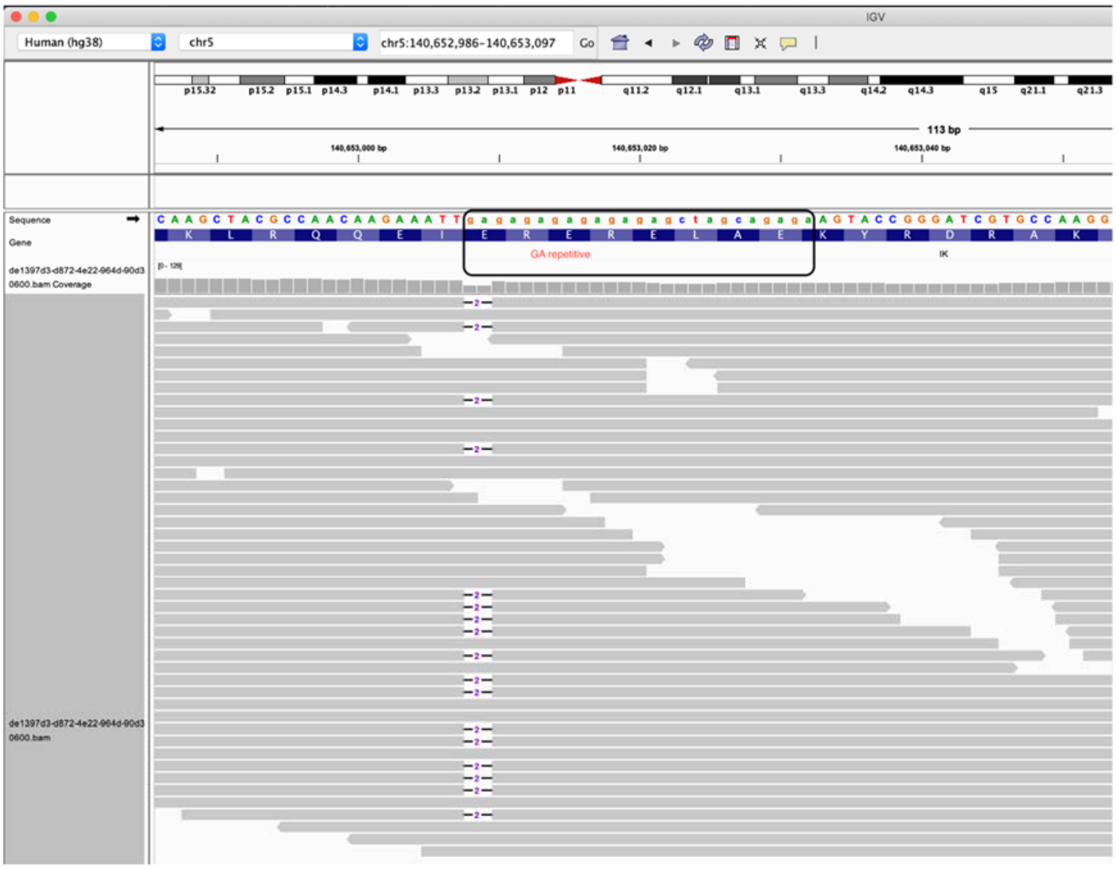

Figure S10. IGV software showed that the length of reads in the alignments is much longer than that of the GA repetition.
